# Effects of High-Intensity Interval Training with Change of Direction Versus Small-Sided Games on Physical Fitness in School-Aged Children

**DOI:** 10.3390/children12091124

**Published:** 2025-08-26

**Authors:** Elzan Bibić, Dušan Stupar, Nebojša Mitrović, Dajana Zoretić, Nebojša Trajković

**Affiliations:** 1 Faculty of Physical Education and Sport, University of East Sarajevo, 71123 East Sarajevo, Bosnia and Herzegovina; elzanbibic9@gmail.com; 2 Faculty of Sport and Psychology, TIMS, Educons University, 21000 Novi Sad, Serbia; dusan.stupar@tims.edu.rs; 3 Faculty of Education, University of East Sarajevo, 76300 Bijeljina, Bosnia and Herzegovina; nebojsa.mitrovic@pfb.ues.rs.ba; 4 Faculty of Kinesiology, University of Zagreb, 10110 Zagreb, Croatia; dajana.zoretic@kif.unizg.hr; 5 Faculty of Sport and Physical Education, University of Nis, 18000 Nis, Serbia

**Keywords:** high intensity, football, physical fitness, adolescents

## Abstract

Background: This study examined the effects of high-intensity interval training with change of direction (HIITcod) and small-sided games (SSGs) on components of physical fitness in school-aged children. The aim was to provide practical insights for optimizing exercise interventions in constrained indoor environments. Methods: A randomized controlled trial was conducted during regular physical education (PE) classes in a school’s indoor sports hall. Fifty healthy boys (mean ± SD = 12.6 ± 0.6 years) were randomly assigned to a HIITcod group (*n* = 25) or an SSG group (*n* = 25). The intervention lasted eight weeks and consisted of structured training sessions designed to progressively increase intensity and training load in a child-friendly and safe environment. Individual maximal heart rate (HRmax) was determined using the 20 m shuttle run test prior to the intervention. Heart rate monitors were worn throughout all sessions to ensure exercise intensity consistently exceeded 75% of HRmax, with real-time monitoring used to adjust effort when necessary. Physical fitness outcomes, including the shuttle run test (SRT), handgrip strength (HG), 20 m sprint, standing broad jump (SBJ), Illinois agility test, and T-test, were assessed pre- and post-intervention. Results: Both groups demonstrated significant improvements over time in the SRT, SBJ, Illinois agility test, and T-test (*p* < 0.05). No significant group × time interactions were detected (all *p* > 0.05). Handgrip strength increased significantly in the HIITcod group (35.03 ± 7.19 kg to 36.80 ± 6.68 kg, *p* = 0.001, d = 0.25) and showed a non-significant trend in the SSG group (38.28 ± 9.09 kg to 39.23 ± 9.12 kg, *p* = 0.056). No significant changes were observed in 20 m sprint performance. Conclusions: Based on the study results, both HIITcod and SSGs were associated with improvements in multiple components of physical fitness in school-aged boys. These findings suggest that both training modalities may be viable options for implementation during physical education classes, particularly in limited indoor settings. The observed positive changes in fitness could indicate their potential to positively impact children’s fitness in a structured and engaging manner.

## 1. Introduction

In recent decades, there has been a concerning decline in physical activity (PA) among children worldwide [1]. Sedentary lifestyles, increased screen time, and reduced opportunities for active play have contributed to deteriorating health outcomes, including obesity, cardiovascular risk factors, and poor motor development [2,3]. In addition to declining PA levels, there is also clear evidence of a decline in physical fitness, particularly in cardiorespiratory fitness, among children and adolescents over the past several decades [4]. Encouragingly, schools present a vital setting to promote physical activity, as they reach children during formative years and can influence lifelong health behaviors through well-designed exercise programs. A comprehensive review of school-based interventions demonstrates that integrative interventions that include a variety of strength and conditioning activities seem to be a promising strategy to promote muscular fitness among youth, supporting the role of schools in shaping long-term behavior [5]. Therefore, implementing effective, enjoyable, and accessible physical activity interventions in school environment may be crucial to combat the trend of inactivity and foster healthier future generations.

Among school-aged children, high-intensity interval training (HIIT) and small-sided games (SSGs) have emerged as efficient and effective interventions for improving multiple health outcomes [6,7,8,9]. HIIT, characterized by brief bursts of intense exercise followed by recovery periods, improves cardiorespiratory fitness and metabolic health markers [6,8,9]. Similarly, SSGs, which replicate the tactical and technical elements of sports in a condensed format, enhance aerobic capacity, agility, coordination, and enjoyment [7,8,10]. Both modalities are practical, adaptable, and highly motivating for children, making them suitable for school fitness programs [9,10]. Notably, combining HIIT and SSGs has even demonstrated additive effects on cardiometabolic health in youth, especially among those with overweight or obesity [8].

Within HIIT approaches, the inclusion of change-of-direction (COD) drills has particular relevance for youth. COD tasks involve repeated acceleration, deceleration, and re-acceleration, imposing greater neuromuscular and metabolic demands compared with linear running [11,12]. These multidirectional actions stimulate agility, balance, and coordination, fundamental motor skills that are critical during childhood and adolescence [13]. Moreover, COD-based HIIT enhances eccentric strength and dynamic stability, factors known to support injury prevention and improve transferability to sport-specific actions. Furthermore, COD movements elevate cardiovascular and metabolic stress beyond that of straight-line running [11], reinforcing their classification as high-intensity activities. Importantly, COD-based HIIT protocols can be executed in limited indoor spaces, making them particularly suitable for school settings where access to large outdoor fields may be restricted. Collectively, these features highlight HIITcod as both developmentally appropriate and environmentally feasible for school-aged children.

Despite accumulating evidence on the benefits of both HIIT and SSGs for children, several critical gaps remain. Most existing school-based HIIT protocols have employed linear running formats, which may not fully capture the multidirectional demands of natural play or sport-specific actions. COD-HIIT, although potentially more developmentally appropriate and feasible in limited indoor spaces, has been insufficiently studied in youth populations. Furthermore, while SSGs are widely recognized for their ecological validity and motivational value, little is known about how their effects compare directly with COD-HIIT on specific health- and performance-related fitness outcomes. Finally, there is a lack of controlled trials that address the practical constraints of school settings, where space limitations often restrict exercise options. Therefore, this study addresses these gaps by directly comparing the effects of COD-HIIT and football-based SSGs on multiple components of physical fitness in school-aged boys, providing valuable insights to optimize exercise interventions within limited indoor spaces.

## 2. Materials and Methods

### 2.1. Participants

This study was designed as a randomized controlled trial. The study was conducted in a school indoor sports hall during regular physical education (PE) classes as part of a structured physical activity intervention. An initial testing session was carried out before the program, followed by a final testing session after the eight-week intervention. The total sample included 50 healthy school children (boys), aged 12 years (mean ± SD = 12.6 ± 0.6), who were randomly assigned to two equal groups: a high-intensity interval training group with change of direction (HIITcod) (*n* = 25) and a small-sided games group (SSG) (*n* = 25) (Table 1). The sample size of 50 participants was determined based on the number of students in available physical education classes at the collaborating school. Due to the study’s pragmatic design and the logistical constraints of conducting the intervention within a real-world school setting, a formal a priori power calculation was not conducted. Biological maturity was calculated for each participant using the formula established by Mirwald et al. [14]. It is a non-invasive method that evaluates the time of greatest increase in height (PHV), taking into account anthropometric characteristics (body height, sitting height, and leg length) and chronological age.

Inclusion criteria were as follows:

(1) Chronological age of 11–13 years;

(2) Regular attendance in school PE classes;

(3) No contraindications for moderate-to-vigorous physical activity, as confirmed by parental health declaration or school medical record.

Exclusion criteria included the following:

(1) Any recent injury or chronic condition preventing physical exertion;

(2) Failure to attend at least 80% of exercise program sessions;

(3) Participation in additional organized sport activities that might influence fitness test results.

**Figure 1:**
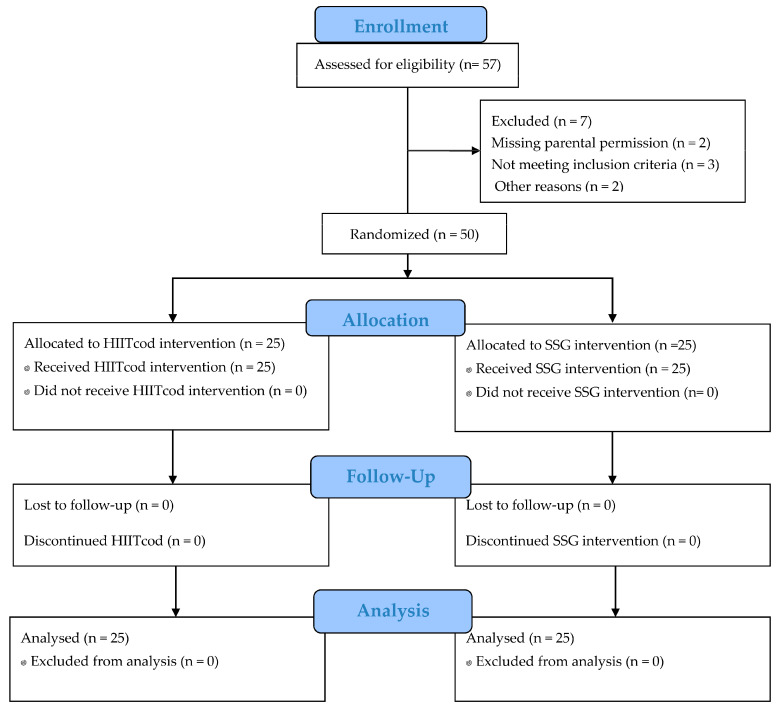
Consolidated Standards of Reporting Trial (CONSORT) guidelines flow diagram.

All procedures were explained to the children and their parents or guardians. Written informed consent was obtained from parents, and verbal assent was given by the children. Moreover, a Consort flow diagram illustrating the recruitment, allocation, follow-up, and analysis of study participants is presented in the Figure 1. The study followed the ethical standards outlined in the Declaration of Helsinki for research involving human participants. The study was approved by the Faculty of sport, the University of Niš, Serbia (decision No. 8/18-01-007/23-031; approval date 6 November 2023).

### 2.2. Procedure

All participants underwent two identical testing sessions—before and after the intervention—under standardized conditions. Training sessions and the majority of physical fitness testing were conducted in the school’s indoor sports hall, while the 20 m shuttle run test (beep test) was performed outdoors on the school field. Children wore appropriate indoor footwear for gym activities and standard PE clothing. Prior to testing, a standardized warm-up consisting of 5 min of light aerobic activity and dynamic stretching was performed.

Testing included anthropometric assessments and physical fitness tests, administered by qualified PE teachers and exercise scientists familiar with youth populations. Due to logistical constraints within the school setting, a standardized testing order was used for all participants to ensure consistency. While a fixed order may introduce order effects—where performance on later tests could be influenced by fatigue from earlier tests—this approach was necessary to accommodate the large number of participants and limited testing time. To mitigate this potential confound, all tests were administered with ample rest periods between each, and the same order was used for all participants both before and after the intervention to allow for a consistent comparison.

#### 2.2.1. Anthropometric Measurements

Anthropometric assessments were performed before motor testing.

- Body height was measured using a portable stadiometer accurate to 0.1 cm.
- Body weight was measured using a digital scale accurate to 0.1 kg.
- Body mass index (BMI) was calculated as weight (kg) divided by height in meters squared (kg/m^2^).

All measurements were conducted barefoot and in minimal PE clothing to ensure accuracy.

#### 2.2.2. Physical Fitness Tests

Physical fitness performance was evaluated using the following standardized tests:

##### 20 m Shuttle Run Test (Beep Test)

This test, conducted outside on the school field, assessed cardiorespiratory endurance. Participants ran back and forth between two lines 20 m apart in time with recorded beeps. The initial speed was 8.5 km/h and increased by 0.5 km/h each minute. Children continued until they failed to reach the line for two consecutive beeps. The last completed stage was recorded to estimate VO_2_max using validated prediction formulas. However, distance covered in meters was used for further analysis.

##### Handgrip Strength Test

Handgrip strength was assessed using a Takei digital handgrip dynamometer (Takei Scientific Instruments Co., Ltd., Niigata, Japan), a valid and reliable tool for evaluating isometric upper-body strength in children and adolescents. The test was conducted indoors in the school gymnasium under standardized conditions.

Each participant was tested individually, standing upright with arms fully extended alongside the body, but not touching the body. The dynamometer’s grip span was adjusted individually to fit the hand size of each participant, ensuring that the second knuckle of the fingers rested comfortably on the handle.

Participants were instructed to squeeze the handle as hard as possible for 3–5 s using their dominant hand without bending the elbow or rotating the trunk. Verbal encouragement was provided to ensure maximal effort. After the first trial, a 30 s rest interval was given, followed by a second trial. The best score, recorded in kilograms (kg) and displayed on the digital screen, was used for analysis.

The procedure was explained and demonstrated before testing, and the children were allowed one practice trial to familiarize themselves with the device. The test has been shown to be age appropriate, safe, and predictive of overall muscular fitness in school-aged children.

##### Standing Long Jump

To assess lower-body explosive strength, participants stood behind a marked line and jumped forward as far as possible using a two-footed take-off. The longest distance in centimeters from the take-off line to the nearest heel mark was recorded from two trials.

##### 20 m Sprint Test

This test evaluated linear sprinting speed. Two timing gates were positioned 20 m apart. From a standing start, participants sprinted through the gates. Time was recorded automatically as they crossed the start and finish lines. Each child completed two trials, with the best result used for analysis.

##### Illinois Agility Test

This test evaluated change-of-direction speed and agility. Eight cones were arranged to create a standardized Illinois layout (10 m × 5 m area). Participants completed the test from a prone position at the start, weaving through cones in a set pattern. Time was recorded with photocell gates.

##### T-Test

The T-test measured multidirectional agility, including sprinting, lateral shuffling, and backpedaling. The test was set up with four cones in a T-shape. Timing began from a standing start and stopped when the participant returned to the start point after completing the required movements. The best out of two attempts in seconds was recorded.

### 2.3. Training Interventions

The participants were randomly assigned to one of two intervention groups: a high-intensity interval training with change of direction (HIITcod) group or a small-sided games (SSGs) group. Both groups participated in the same standard PE curriculum appropriate for their age group, in accordance with national educational guidelines. However, in place of the regular main activity segment, one group performed the HIITcod program while the other engaged in SSG activities. The interventions were implemented as part of regularly scheduled PE classes, conducted twice per week over an eight-week period in the school’s indoor sports hall. In Serbia, PEis mandatory, with 2 to 3 classes per week depending on the student’s age. In this study, all participants had two obligatory 45 min PE classes and one additional class of mandatory physical activity; however, due to the organizational structure of the intervention, the training programs were implemented during the two obligatory PE classes each week. The intervention sessions were closely supervised by both the researcher and a physical education specialist to ensure proper execution and participant safety. Maximal heart rate (HRmax) was determined individually for each participant using the 20 m shuttle run test conducted prior to the intervention. While direct measurements of VO_2_max was not feasible in this school-based setting, the shuttle run test provided a valid estimation of aerobic fitness and allowed for individualized HRmax prescription. To ensure that exercise intensity consistently exceeded 75% of HRmax, heart rate monitors were used throughout the entire eight-week intervention period. Continuous monitoring allowed for real-time tracking and adjustment of effort during each session, ensuring that participants maintained the targeted intensity level across all training weeks. The average heart rates during the intervention were similar between the HIITcod (172.1 ± 7.3 bpm) and SSG (167.0 ± 6.14 bpm) groups, indicating comparable training intensity across both protocols. Because of the indoor space limitations, both interventions emphasized frequent changes of direction, and the SSGs were restricted to 2v2 and 3v3 formats on appropriately scaled areas (e.g., 10 × 12 m for 2v2 and 15 × 18 m for 3v3). Each session began with a 10 min group warm-up (mobility drills, dynamic stretching, and progressive runs) and concluded with a 5 min cool-down (light jogging and static stretching), making the total session last 45 min.

### 2.4. HIIT with Change of Direction (HIITcod)

The HIITcod program consisted of short running intervals of 15 s followed by 15 s of passive recovery (standing or walking). Each 15 s run was performed at a self-paced but vigorous intensity, encouraging participants to reach near-maximal effort while completing multiple 180° turns within each run. Due to the indoor setting, each run included 3 to 5 changes of direction, which increased neuromuscular and cardiovascular demands and was appropriate for the available space.

Training loads were progressively increased across the 8-week period by manipulating the number of repetitions and sets while ensuring that the main training part lasted approximately 30 min. For example, early sessions began with 4 sets of 6 repetitions (24 intervals = 12 min total work + rest) and progressed to 5 sets of 12 repetitions (60 intervals = 30 min total) by week 8, all while maintaining the 15:15 work-to-rest ratio. Cones were used to set turning points at 3–5 m intervals, adjusted to the participants’ ability and hall dimensions.

#### Small-Sided Games Group (SSG)

The SSG group engaged in game-based conditioning using 2v2 and 3v3 formats appropriate for the indoor hall dimensions. Games were played on small courts (approx. 10 × 12 m for 2v2 and 15 × 18 m for 3v3), with rules adapted to maintain high intensity and constant engagement. Each session included 4 to 5 bouts of 4–5 min, with 2 min passive recovery periods between bouts, totaling approximately 30 min of training.

Game formats alternated weekly to promote technical variety and motivation, while keeping sessions fun and developmentally appropriate. No formal tactical instructions were provided during play to encourage free movement and spontaneous decision making, with an emphasis on maximizing aerobic load, agility, ball control, and enjoyment. The interventions were designed to comply with current recommendations for physical activity in school children and to promote enjoyment alongside physical development.

### 2.5. Statistical Analysis

All statistical analyses were conducted using IBM SPSS Statistics version 23.0 (IBM Corp., Armonk, NY, USA). Descriptive statistics were calculated for all variables and presented as means and standard deviations. The assumption of normality was assessed using the Shapiro–Wilk test. To examine baseline differences between groups, independent samples t-tests were performed for each dependent variable. To evaluate the effects of the intervention over time and between training groups, a two-way repeated measures ANOVA (time × group) was conducted. Where significant interaction effects were found, Bonferroni post hoc tests were applied to identify within- and between-group differences. An alpha level of *p* < 0.05 was considered statistically significant.

Moreover, effect size (ES) with confidence intervals was included with ES classified as follows: <0.2 was defined as trivial; 0.2–0.6 was defined as small; 0.6–1.2 was defined as moderate; 1.2–2.0 was defined as large; >2.0 was defined as very large; and >4.0 was defined as extremely large.

## 3. Results

All data passed the normality tests. General descriptive statistics for both groups are shown in Table 1. Participants were aged 11 to 13 years (M = 12.6 ± 0.6), with biological maturity (Y-PHV = 1.3 ± 0.8), which estimates time to and from peak height velocity using anthropometric measurements. Age, body height, body mass, and BMI were comparable between groups (*p* > 0.05), with small effect sizes, indicating similar baseline profiles and appropriate group comparability before the intervention.

Independent t-tests revealed no significant baseline differences between the HIITcod and SSG groups in any performance variables (all *p* > 0.05). Repeated-measures ANOVA revealed no significant group × time interaction for any of the tested variables (all *p* > 0.05), indicating that both interventions led to comparable improvements across the performance outcomes (Table 2).

Despite the lack of interaction effects, significant time effects were observed in both groups. For the shuttle run test (SRT), the HIITcod group improved from 535.3 ± 199.4 m to 657.3 ± 205.2 m (*p* = 0.001), while the SSG group improved from 518.7 ± 117.9 m to 604.0 ± 136.9 m (*p* = 0.02). Both groups also improved in handgrip strength (HG), with the HIITcod group increasing from 35.0 ± 7.2 kg to 36.8 ± 6.7 kg (*p* = 0.001) and the SSG group from 38.3 ± 9.2 kg to 39.2 ± 9.1 kg (*p* = 0.05). Although statistically significant, the effect sizes were small in both groups (HIITcod: d = 0.25, 95% CI: −0.26 to 0.76; SSG: d = 0.10, 95% CI: −0.41 to 0.61), indicating limited practical relevance of the observed changes.

In lower-body explosive power, both groups significantly increased their standing broad jump (SBJ) distances. The HIITcod group improved from 157.1 ± 19.7 cm to 168.6 ± 17.1 cm (*p* = 0.020) and the SSG group from 152.9 ± 19.0 cm to 161.7 ± 16.5 cm (*p* = 0.041). Agility tests also revealed meaningful improvements. The Illinois agility test scores decreased significantly in both groups (HIITcod: *p* = 0.012; SSG: *p* = 0.044), as did the T-test scores (HIITcod: *p* = 0.001; SSG: *p* = 0.02), reflecting better agility performance.

No significant changes were observed in 20 m sprint performance in either group (*p* > 0.05), suggesting that this particular fitness attribute was less responsive to the training interventions used.

## 4. Discussion

This study aimed to compare the effects of high-intensity interval training with change of direction (HIITcod) and small-sided games (SSGs) on health-related physical fitness in school-aged boys within an indoor school setting. Over the course of the eight-week intervention, both groups improved significantly in cardiorespiratory fitness, lower-body power, agility, and handgrip strength, while no group × time interaction effects were observed. Importantly, neither intervention influenced linear sprint performance.

Cardiorespiratory fitness (CRF), assessed via the shuttle run test (SRT), increased in both groups (HIITcod: +122.0 m; SSG: +85.3 m). These results corroborate systematic reviews and empirical evidence demonstrating that both HIIT and SSG are effective in enhancing CRF in youth [6,7,8]. The slightly larger gain in the HIITcod group, although not statistically significant, is consistent with previous work showing that HIIT elicits greater cardiovascular benefits when intensity is objectively monitored to exceed ~75% HRmax [9,15]. In the present study, heart rate telemetry confirmed intensity control in HIITcod sessions, whereas intensity in SSG was not directly regulated. This methodological distinction may partly explain the trend toward greater CRF improvements in the HIITcod group, highlighting the importance of objective intensity monitoring in school-based interventions.

Muscular strength, assessed via handgrip performance, also improved in both groups. The HIITcod group demonstrated a statistically significant increase (from 35.03 ± 7.19 kg to 36.80 ± 6.68 kg, *p* = 0.001), while the SSG group showed a non-significant but positive trend (from 38.28 ± 9.09 kg to 39.23 ± 9.12 kg, *p* = 0.056). These findings are consistent with research suggesting that HIIT incorporating multidirectional running and bodyweight resistance can enhance strength [5,16]. Interestingly, the improvement observed in SSG suggests that the dynamic, whole-body nature of gameplay provides sufficient muscular loading to stimulate adaptations, even in the absence of targeted resistance training [17]. Our findings also align with those of Jovanović et al. [18], who reported that school-based HIIT improved muscular fitness in adolescents.

Explosive power, as measured by the standing broad jump (SBJ), improved in both groups (HIITcod: +11.51 cm; SSG: +8.78 cm), confirming previous findings that structured HIIT and SSG protocols stimulate neuromuscular adaptations in youth [15,17]. Improvements in agility, demonstrated by reduced times in the Illinois agility test and the T-test, reinforce the value of incorporating multidirectional movement patterns into PE-based interventions [8].

By contrast, no significant improvements were observed in 20 m sprint performance. This is consistent with prior research showing that interventions emphasizing agility and endurance elicit limited transfer to linear sprint performance [19]. As sprint acceleration and maximal velocity rely on specific neuromechanical factors, targeted sprint drills would be necessary to induce meaningful gains in this domain.

The comparable adaptations across both groups can be attributed to several shared characteristics: repeated high-intensity bouts, frequent changes of direction, and dynamic movement patterns integrated into regular PE classes. These overlapping features may have minimized differences between protocols, suggesting that in school settings, the total dose of vigorous physical activity may be more influential for children’s adaptation than the specific exercise format [5,18]. Furthermore, the restricted indoor environment standardized the external conditions for both groups, potentially reducing variability between interventions.

Collectively, these findings extend the prior literature by demonstrating that both HIITcod—with objectively controlled intensity—and SSG—with ecological, game-based engagement—are feasible and effective for improving multiple fitness components in school-aged boys. The absence of between-group differences underscores the value of both modalities in school PE while also highlighting the need for more rigorously controlled trials to disentangle the relative contributions of intensity regulation, training structure, and enjoyment in shaping youth fitness outcome.

### Limitations and Strengths

This study has several strengths. First, it was conducted as a randomized controlled trial within a real-world school setting, enhancing ecological validity and demonstrating the feasibility of implementing structured high-intensity programs during regular PE classes. Second, exercise intensity in the HIITcod group was objectively monitored using heart rate telemetry, ensuring that participants consistently exceeded the targeted threshold of 75% HRmax. Third, the use of well-established, validated fitness assessments provided reliable measures across multiple physical fitness domains. Finally, the intervention was designed to be developmentally appropriate, space efficient, and aligned with the resources typically available in schools, thereby increasing its translational potential for educational practice.

Nevertheless, some limitations should be acknowledged. First, due to logistical constraints within a school setting, a fixed order was used for the physical fitness tests. This approach did not control for potential order effects, where fatigue from earlier tests could have influenced performance on subsequent tests (e.g., the agility tests). Second, the study sample consisted exclusively of boys aged 12 years, limiting the generalizability of findings to girls or other age groups. Third, despite random assignment, potential maturation-related effects cannot be fully excluded, as participants were close to the period of peak height velocity. Fourth, the relatively small sample size and short intervention duration (eight weeks) may have limited the detection of between-group differences and long-term adaptations. However, the sample size was limited by the number of students available in the participating school, and a formal a priori power calculation was not conducted, which may have limited the statistical power to detect smaller between-group differences. Finally, participation was restricted to a single school, which may introduce selection bias and limit external validity.

## 5. Conclusions and Practical Application

The present randomized controlled trial indicates that both HIITcod and SSGs, when implemented within physical education classes in indoor school settings, were associated with improvements in cardiorespiratory fitness, muscular strength, power, and agility in school-aged boys. No significant differences between interventions were observed, suggesting that both approaches represent feasible options for promoting health-related physical fitness in space-constrained school environments.

From a practical perspective, these findings support the use of structured, high-intensity activities such as HIITcod and SSGs as complementary strategies in physical education curricula, particularly in contexts where access to large outdoor facilities is limited. Importantly, both interventions appear to be developmentally appropriate, time efficient, and adaptable to the resources typically available in schools. Future research should explore the long-term sustainability of these adaptations, include female participants and diverse age ranges, and investigate additional health and psychosocial outcomes such as motivation, enjoyment, and adherence. Such evidence will help to refine recommendations for school-based programs aimed at improving physical fitness and overall well-being in youth.

## Figures and Tables

**Table 1:** Baseline characteristics of participants in the HIITcod and SSG groups.

Variable	HIITcod (Mean ± SD)	SSG (Mean ± SD)	*p*-Value	Cohen’s d	95% CI for Difference
Age (years)	12.5 ± 0.6	12.7 ± 0.6	0.265	0.33	−0.23 to 0.89
Body height (cm)	171.2 ± 6.3	170.1 ± 6.7	0.511	0.17	−0.39 to 0.73
Body mass (kg)	62.8 ± 6.9	62.0 ± 7.5	0.723	0.11	−0.44 to 0.67
BMI (kg/m^2^)	21.4 ± 2.0	20.9 ± 2.1	0.434	0.24	−0.32 to 0.80

Note: BMI—body mass index.

**Table 2:** Changes in physical fitness in HIITcod and SSG groups.

Test	HIITcod Pre (Mean ± SD)	HIITcod Post (Mean ± SD)	*p*	ES (95% CI)	SSG Pre (Mean ± SD)	SSG Post (Mean ± SD)	*p*	ES (95% CI)
SRT (m)	535.3 ± 199.4	657.3 ± 205.2	0.001	0.6 (0.05, 1.15)	518.7 ± 117.9	604.0 ± 136.9	0.02	0.6 (0.05, 1.15)
HG (kg)	35.0 ± 7.2	36.8 ± 6.7	0.001	0.25 (−0.26, 0.76)	38.3 ± 9.2	39.2 ± 9.1	0.05	0.1 (−0.41, 0.61)
Sprint 20 m (s)	4.5 ± 0.3	4.4 ± 0.2	0.17	−0.1 (−0.41, 0.61)	4.4 ± 0.2	4.4 ± 0.4	1.00	0 (−0.51, 0.51)
SBJ (cm)	157.1 ± 19.7	168.6 ± 17.1	0.02	0.6 (0.05, 1.15)	152.9 ± 19.0	161.7 ± 16.5	0.04	0.5 (−0.04, 1.04)
Illinois test (s)	19.6 ± 0.7	19.4 ± 0.6	0.01	−0.3 (−0.82, 0.22)	19.7 ± 0.6	19.6 ± 0.5	0.04	0.2 (−0.71, 0.31)
T-test (s)	12.6 ± 0.7	12.3 ± 0.5	0.001	−0.4 (−0.97, 0.17)	12.6 ± 0.5	12.5 ± 0.3	0.02	−0.1 (−0.66, 0.46)

SBJ—standing broad jump; SRT—shuttle run test; HG—hand grip dynamometer test. *p*—significant differences between initial and final testing; 95% CI 5—95% confidence interval.

## Data Availability

The data are available on request from the corresponding author.

## References

[1] Aubert S., Barnes J.D., Demchenko I., Hawthorne M., Abdeta C., Nader P.A., Sala J.C.A., Aguilar-Farias N., Aznar S., Bakalár P. (2022). Global Matrix 4.0 Physical Activity Report Card Grades for Children and Adolescents: Results and Analyses from 57 Countries. J. Phys. Act. Health.

[2] LeBlanc A.G., Gunnell K.E., Prince S.A., Saunders T.J., Barnes J.D., Chaput J.P. (2017). The Ubiquity of the Screen: An Overview of the Risks and Benefits of Screen Time in Our Modern World. Transl. J. Am. Coll. Sports Med..

[3] Poitras V.J., Gray C.E., Borghese M.M., Carson V., Chaput J.-P., Janssen I., Katzmarzyk P.T., Pate R.R., Connor Gorber S., Kho M.E. (2016). Systematic Review of the Relationships between Objectively Measured Physical Activity and Health Indicators in School-Aged Children and Youth. Appl. Physiol. Nutr. Metab..

[4] Fühner T., Kliegl R., Arntz F., Kriemler S., Granacher U. (2021). An Update on Secular Trends in Physical Fitness of Children and Adolescents from 1972 to 2015: A Systematic Review. Sports Med..

[5] Villa-González E., Barranco-Ruiz Y., García-Hermoso A., Faigenbaum A.D. (2023). Efficacy of School-Based Interventions for Improving Muscular Fitness Outcomes in Children: A Systematic Review and Meta-Analysis. Eur. J. Sport Sci..

[6] Bauer N., Sperlich B., Holmberg H.C., Engel F.A. (2022). Effects of High-Intensity Interval Training in School on the Physical Performance and Health of Children and Adolescents: A Systematic Review with Meta-Analysis. Sports Med. Open.

[7] Gómez-Álvarez N., Boppre G., Hermosilla-Palma F., Reyes-Amigo T., Oliveira J., Fonseca H. (2024). Effects of Small-Sided Soccer Games on Physical Fitness and Cardiometabolic Health Biomarkers in Untrained Children and Adolescents: A Systematic Review and Meta-Analysis. J. Clin. Med..

[8] Zheng B., Xu Q., Zhang J. (2025). Combining HIIT with Small-Sided Soccer Games Enhances Cardiometabolic and Physical Fitness More Than Each Alone in Overweight Youth: A Randomized Controlled Study. J. Sports Sci. Med..

[9] Lubans D.R., Eather N., Smith J.J., Beets M.W., Harris N.K. (2022). Scaling-Up Adolescent High-Intensity Interval Training Programs for Population Health. Exerc. Sport Sci. Rev..

[10] Clemente F.M., Martinho D.V., Silva R., Trybulski R., Sánchez-Sánchez J., Rodríguez-Fernández A., Beato M., Afonso J. (2025). Physiological, Physical and Technical Demands during Sided Soccer Game Formats: A Review. Int. J. Sports Med..

[11] Hader K., Palazzi D., Buchheit M. (2016). Change-of-Direction Speed in Soccer: How Much Braking Is Enough?. Int. J. Sports Physiol. Perform..

[12] Sheppard J.M., Young W.B. (2006). Agility Literature Review. J. Sports Sci..

[13] Faigenbaum A.D., Lloyd R.S., Myer G.D. (2013). Youth resistance training: Past practices, new perspectives, and future directions. Pediatr. Exerc. Sci..

[14] Mirwald R.L., Baxter-Jones A.D., Bailey D.A., Beunen G.P. (2002). An Assessment of Maturity from Anthropometric Measurements. Med. Sci. Sports Exerc..

[15] Duncombe S.L., Barker A.R., Bond B., Earle R., Varley-Campbell J., Vlachopoulos D., Walker J.L., Weston K.L., Stylianou M., Harnish C. (2022). School-Based High-Intensity Interval Training Programs in Children and Adolescents: A Systematic Review and Meta-Analysis. PLoS ONE.

[16] Mitić P., Jovanović R., Stojanović N., Barišić V., Trajković N. (2024). Enhancing Adolescent Physical Fitness and Well-Being: A School-Based High-Intensity Interval Training Program. J. Funct. Morphol. Kinesiol..

[17] Petrušič T., Trajković N., Bogataj Š. (2022). Twelve-Week Game-Based School Intervention Improves Physical Fitness in 12–14-Year-Old Girls. Front. Public Health.

[18] Jovanović R., Živković M., Stanković M., Zoretić D., Trajković N. (2024). Effects of School-Based High-Intensity Interval Training on Health-Related Fitness in Adolescents. Front. Physiol..

[19] Jurić P., Dudley D.A., Petocz P. (2023). Does Incorporating High Intensity Interval Training in Physical Education Classes Improve Fitness Outcomes of Students? A Cluster Randomized Controlled Trial. Prev. Med. Rep..

